# Fear of progression profiles in parents of children with cancer and their impact on psychological distress: The mediating role of sense of coherence

**DOI:** 10.1016/j.apjon.2026.100926

**Published:** 2026-03-03

**Authors:** Guiyuan Ma, Peijuan Jiao, Xiaorou Zeng, Qiming Ding, Zhixiang Huang, Can Gu, Jianhui Xie

**Affiliations:** a Xiangya School of Nursing, Central South University, Changsha, China; b The Children's Hospital Affiliated to Xiangya School of Medicine, Central South University (Hunan Children's Hospital), Changsha, China

**Keywords:** Children with cancer, Parents, Fear of progression, Sense of coherence, Psychological distress, Latent profile analysis

## Abstract

**Objective:**

To identify latent profiles of Fear of Progression (FoP) in parents of children with cancer, explore their associated factors, and test the mediating role of Sense of Coherence (SOC) between FoP and psychological distress (PD).

**Methods:**

A cross-sectional study was conducted with 273 parents of children with cancer in China. We used latent profile analysis (LPA) to identify FoP profiles, multinomial logistic regression to determine associated factors, and mediation analysis to test the role of SOC.

**Results:**

Three distinct FoP profiles were identified: medication sensitive with low fear (38%), treatment sensitive with moderate fear (21%), and overall high fear (41%). These profiles were significantly differentiated by disease-related (e.g., treatment history), individual-related, and interpersonal-related (e.g., self-disclosure) factors. Across the sample, higher FoP was associated with greater PD. Importantly, mediation analyses revealed that SOC significantly mediated the relationship between FoP and PD for the moderate and high FoP profiles, but not for the low LoP profile.

**Conclusions:**

Parents of children with cancer exhibit heterogeneous FoP profiles. SOC acts as a crucial mediator between FoP and PD, particularly for parents with moderate and high FoP profiles. These findings underscore the importance of screening for specific FoP profiles and suggest that tailored interventions designed to enhance SOC could effectively reduce PD in high-risk parents.

## Introduction

Childhood cancer remains one of the most serious diseases affecting children worldwide,^1^ with a steadily increasing incidence in recent years. Epidemiological data from the World Health Organization estimate that between 275,000 and 400,000 children are diagnosed with cancer each year worldwide.^2^ Although advances in medical technology have gradually improved overall survival rates in pediatric cancer,^3^, ^4^, ^5^ the disease and its prolonged treatment continue to impose substantial physical and psychological burdens on affected children, while also generating heavy psychological and financial strain on their families.^4^, ^5^, ^6^, ^7^, ^8^ In China, the primary caregivers of pediatric patients are typically their parents, who are not only responsible for complex caregiving tasks but also face prolonged uncertainty and risk of relapse.^9^^,^^10^ As a result, parents of children with cancer represent a particularly vulnerable group in terms of psychological adjustment.^11^^,^^12^

Fear of progression (FoP)^13^, ^14^, ^15^ refers to a persistent worry and fear related to the possibility of disease recurrence or progression in the context of chronic illness or cancer.^14^ Accumulating evidence indicates that sustained FoP among parents of children with cancer is associated with psychological distress (PD), including chronic anxiety, depression, sleep disturbances, and impaired caregiving capacity.^16^^,^^17^ Studies have shown that FoP is one of the most common psychological problems among cancer patients and their family members.^16^^,^^18^^,^^19^

Most existing FoP studies have focused on adult cancer patients. For instance, Goebel et al. reported that 42% of adult brain cancer patients experienced high FoP;^7^ Zhuang et al. found that FoP scores among breast cancer patients were 35.87 ± 9.24;^18^ and Li et al. reported a FoP score of 32.94 ± 10.64 in patients with gastrointestinal cancers^20^ (total score ≥ 34 indicating a high level of FoP).^21^ Research specifically targeting parents of children with cancer is relatively limited. Available studies suggest a high prevalence of FoP in this population; for instance, Yu et al. found that 68.1% of parents of children with retinoblastoma experienced FoP-related functional impairment.^6^ However, these studies have largely conceptualized FoP as a homogeneous construct,^16^ overlooking potential heterogeneity in FoP manifestations among parents and limiting the development of tailored interventions.^6^^,^^16^^,^^22^

Latent profile analysis (LPA) is a person-centered statistical approach that identifies unobserved subgroups based on response patterns across multiple indicators.^22^ Compared with traditional variable-centered approaches, LPA allows for the identification of qualitatively distinct psychological profiles within heterogeneous populations.^23^^,^^24^ Liu et al. have identified three latent FoP profiles among Chinese survivors of hematologic malignancies.^25^ However, cancer survivors differ substantially from parents of children, who face unique challenges related to caregiving responsibilities, long-term prognosis concerns, and family adaptation.^16^^,^^26^ Thus, the latent structure of FoP in parents of children with cancer remains unclear and warrants further investigation.^22^

Beyond identifying FoP profiles, clarifying the psychological mechanisms linking FoP to PD across different profiles is essential for intervention development. According to Lazarus and Folkman's transactional model of stress and coping,^27^ individual's cognitive appraisal and perceived resources shape their emotional responses to stress.^27^ Within this framework, sense of coherence (SOC)^28^ has been conceptualized as a key psychological resource that explains how individuals comprehend and manage ongoing stress. SOC comprises three components: comprehensibility, manageability, and meaningfulness, which reflect how individuals cognitively process events and orient their motivations in the face of adversity.^28^^,^^29^ For parents of children with cancer, higher SOC are better able to understand disease-related information, perceive a sense of control over their lives and their child's condition, and derive meaning from caregiving experiences.^29^ Previous studies have shown that SOC often plays a crucial role between stress and PD;^30^^,^^31^ for example, among advanced lung cancer and spousal caregivers, higher SOC is associated with lower psychological burden.^31^ However, in the population of parents of children with cancer, the role of SOC in modulating the relationship between FoP and PD remains insufficiently explored.

Therefore, this study focuses on parents of children with cancer and employs a cross-sectional design to: (1) identify latent FoP profiles among parents; (2) compare disease-related, individual-related, interpersonal-related and organizational-related factors across the different FoP profiles based on the socio-ecological model;^32^ and (3) test the mediating role of SOC in the relationship between FoP and PD according to the transactional stress model.^27^ By elucidating heterogeneity and underlying mechanisms of FoP, this study aims to provide empirical evidence to support the identification of high-risk parent groups and the development of targeted interventions.

## Methods

### Participants

This cross-sectional study was conducted between September and November 2025 in the pediatric-oncology departments of three provincial hospitals in Changsha, Hunan Province, China. Participants were parents of children with cancer, recruited using a convenience sampling method.

Inclusion criteria for the parents were as follows: (1) being a biological or legal guardian aged ≥ 18 years; (2) serving as the primary caregiver for a child with cancer for at least the preceding three months, defined as being the person primarily responsible for the child's daily care and medical decision-making;^13^ (3) having adequate Chinese literacy to complete the questionnaire independently; and (4) providing written informed consent.

Furthermore, parents were eligible to participate only if their child met the following criteria: (1) aged between 0 and 18 years; (2) had a confirmed diagnosis of cancer; and (3) was currently undergoing active inpatient anticancer treatment. Parents were excluded if their child had severe comorbid systemic diseases, inherited metabolic disorders, or an unstable clinical condition, in order to minimize confounding influences related to critical illness unrelated to the cancer diagnosis and treatment.

### Sample size

This study employed convenience sampling. Based on previous research, the calculation of LPA sample size primarily relies on three rules: (1) Each profile should contain at least 50 samples;^33^ (2) The smallest profile should comprise at least 5% of the total sample;^23^ and (3) The total sample size should be at least 5 to 10 times the number of independent variables, accounting for a 20% attrition or dropout rate.^24^ In this study, we proposed 23 independent variables, implying a minimum requirement of 144–288 samples. [23 × (5–10) / (1% to 20%) = 144 to 288]. Our 273 participants met these requirements while also satisfying the criteria of at least 50 samples per subgroup and a class probability of at least 5%, indicating that the latent profiles we identified are stable and interpretable.

### Measurement

Based on a literature review and discussions within the research team, relevant factors for measurement were determined according to the socio-ecological model.^32^ As the children were in the inpatient anticancer treatment phase, participants’ main experiences and resources were concentrated within the family and health care settings. Consequently, community- and macro-policy-related factors were less salient at this stage and more difficult to obtain, and therefore were not included. The disease-related, individual-related, interpersonal-related, and organizational-related factors examined in this study are summarized in Table 1. The scale is available in the **Supplementary File 1**.

**Table 1:** Factors collected in this study.

Level	Related factors	Measurement
Disease-related (child)	Diagnosis, treatment duration, chronic diseases/infections/complications, BMI (Body Mass Index), anemia^a^, history of surgery, history of hematopoietic stem cell transplantation, radiotherapy history, chemotherapy cycle, recurrence, CVAD (Central Venous Access Device) catheter	General information questionnaire
Individual-related	Parent's gender, parent's age, child's gender, child's age, residence, educational level^a^, family monthly income (per person), occupation^a^	General information questionnaire
Interpersonal-related	Social support, self-disclosure	PSSS, DDAT
Organizational-related	Perceived communication quality (by doctors/nurses), received social work services (financial assistance), employment status	General information questionnaire

Educational level was categorized as: illiterate; primary school; middle school; high school; technical secondary school; junior college diploma; and bachelor's degree or above.

Occupation was categorized as: employees of state-owned or private enterprises; employees of government or public institutions; farmers; self-employed individuals; retired; unemployed/resigned; and others.

a The anemia means current condition. Anemia reflects the child's current treatment condition and represents a common, observable complication that may amplify parents' perceptions of disease severity and treatment burden.

#### General information questionnaire

The questionnaire collected demographic and clinical information, including diagnosis, duration of treatment, comorbid chronic diseases, infections, complications, body mass index (BMI), and other relevant variables (Table 1).

#### Fear of progression

The Fear of Progression Questionnaire-Short Form for Parents (FoP-Q-SF/PR)^6^^,^^34^ is a self-report instrument developed by Schepper et al., in 2015 and has been widely used to assess the severity of disease progression concerns among parents of children with various illnesses. We used the Chinese version of the FoP-Q-SF/PR, translated and culturally adapted by Yang et al., in 2022.^14^ The Cronbach's alpha coefficient for the total scale in the Chinese population validation was 0.893, the split-half reliability was 0.894, and the test-retest reliability was 0.819.^14^ The scale has includes 12 items. Responses are rated on a 5-point Likert scale from 1 (strongly disagree) to 5 (strongly agree), yielding a total score range of 12–60, with higher scores indicating greater concern about their child's disease progression. Scores above 34 indicate levels exceeding the normal range.^21^ In the present study, Cronbach's alpha coefficient was 0.924, indicating good internal consistency.

#### Psychological distress

The Kessler Psychological Distress Scale (K10) was developed by Kessler et al.^35^^,^^36^ and is widely used to assess non-specific symptoms of anxiety and depression.^35^ In this study, we used the Chinese version of the K10, translated and validated by Zhou et al.,^36^ to evaluate PD experienced by participants. The split-half reliability coefficient of the Chinese version of the K10 scale validated in the Chinese population is 0.708, with a Cronbach's alpha coefficient of 0.801.^36^ The scale consists of 10 items, each rated on a 5-point Likert scale from 1 (none) to 5 (all of the time), with total scores ranging from 10 to 50. According to the total score, PD is categorized into four levels: minimal (10–15), mild (16–21), moderate (22–29), and severe (30–50). In this study, Cronbach's alpha was 0.942.

#### Perceived social support

The Perceived Social Support Scale (PSSS), developed by Zimet et al., in 1990^37^ and translated by Jiang et al.,^38^ was used to assess individuals' perceived social support from multiple sources, such as family, relatives, colleagues, and friends. The Cronbach's alpha coefficient for the Chinese version of the PSSS scale among the Chinese population is 0.832.^38^ The scale consists of 12 items rated on a 7-point Likert scale ranging from 0 (very strongly disagree) to 7 (very strongly agree), with a total score ranging from 12 to 84. Scores of ≤ 36 indicate low perceived support, 37–60 indicate moderate support, and 61–84 indicate high support.^37^^,^^38^ In this study, the scale showed high internal consistency, with a Cronbach's alpha of 0.901.

#### Sense of coherence

The 13-item Sense of Coherence scale (SOC-13), developed by Antonovsky and translated into Chinese by Bao et al.,^29^^,^^39^ was used to assess participants' SOC. The SOC-13 scale demonstrated a Cronbach's alpha coefficient of 0.780 when validated in the Chinese population, with a test-retest reliability of 0.61 and an internal consistency coefficient of 0.76.^39^ The SOC-13 comprises three dimensions: comprehensibility, manageability, and meaningfulness. Items are rated on a 7-point Likert scale, with five items (1, 2, 3, 8, 13) reverse-scored. The total score ranges from 13 to 91, with higher scores indicating a stronger SOC. Scores of 80–91 indicate a high level, 64–79 indicate a moderate level, and 13–63 indicate a low level of SOC. In this study, Cronbach's alpha was 0.865.

#### Distress disclosure

The Distress Disclosure Assessment Tool (DDAT), developed by Kahn et al., is designed to quantify an individual's tendency to share personal distress with others.^37^ We used the Chinese version of the DDAT, translated and revised by Li et al.,^40^ to assess the level of self-disclosure among parents of children with cancer. The Cronbach's alpha coefficient for the Chinese version of the DDAT scale validated in the Chinese population is 0.866, with a split-half reliability coefficient of 0.847.^40^ Items are rated on a 5-point Likert scale, from 1 (strongly disagree) to 5 (strongly agree), yielding a total score range of 12–60, with low distress disclosure at 12–29 points; medium at 30–44 points; and high at 45–60 points.^37^^,^^40^ In this study, Cronbach's alpha was 0.866.

### Data collection and quality control

Prior to study initiation, all investigators underwent a comprehensive, standardized training program. This program covered the study's objectives, detailed inclusion and exclusion criteria, standardized procedures for questionnaire administration, ethical principles including privacy protection, and data entry protocols. A pilot survey involving 20 participants was conducted to evaluate the clarity of the questionnaire items, estimate the completion time (which was determined to be 15–25 minutes), and test the feasibility of the data entry process.

**Fig. 1:**
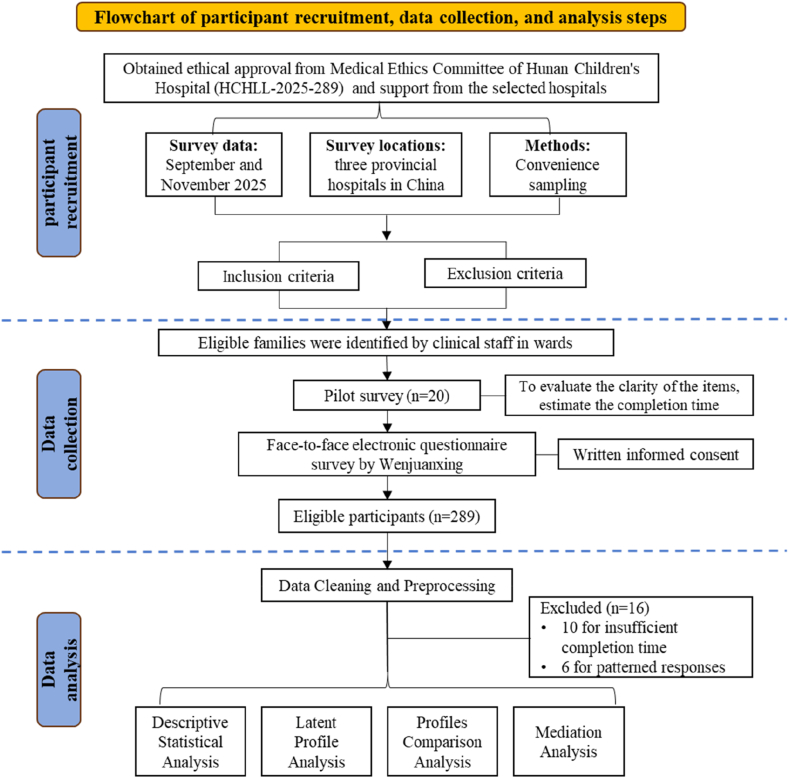
Flowchart of participant recruitment, data collection and data analysis.

During the formal data collection, eligible families were identified by clinical staff in wards and provided informed consent. Data were primarily face-to-face collected via electronic questionnaires on the Wenjuanxing platform, with all items set as mandatory to prevent missing responses. For participants with limited literacy or special conditions preventing self-administration, trained research assistants provided one-on-one support by reading the questionnaire items aloud in a neutral tone. Out of 289 distributed questionnaires, 10 were excluded for insufficient completion time (< 15 minutes) and 6 for patterned responses. After these exclusions, a total of 273 parents completed the survey, yielding a clean dataset with no missing items (Fig. 1).

### Ethical consideration

The study was approved by the Medical Ethics Committee of Hunan Children's Hospital (Approval No. HCHLL-2025-289). Prior to enrollment, all participants provided electronic written informed consent after receiving a detailed explanation of the study's objectives, procedures, potential risks, and anticipated benefits. Consent was obtained by requiring participants to actively confirm their agreement before accessing the questionnaire, and only those who provided consent were allowed to proceed. Participants were informed of their right to withdraw from the study at any time without any impact on their clinical care or treatment. All data collected were anonymized using unique numerical identifiers and securely stored on a password-protected computer accessible only to the research team. This cross-sectional study was conducted in accordance with the Declaration of Helsinki.

### Statistical analysis

Descriptive statistics were performed using SPSS 28.0. Continuous variables were presented as mean ± SD or median (Q1, Q3), and categorical variables as frequencies and percentages. Chi-square or rank-sum tests were used to compare demographic and scale scores across different FoP categories. The Bonferroni method was used for testing differences between profiles. Variables showing significant differences were further analyzed using Logistic regression to identify predictors of FoP profiles. Statistical significance was set at *P* < 0.05 for two-tailed tests.

LPA was conducted in Mplus 8.0 using the 12 items of the FoP-Q-SF/PR as observed indicators. Models with 1–5 classes were compared, and the optimal solution was selected based on fit indices and interpretability. Model fit was assessed using: (1) information criteria: Akaike Information Criterion (AIC), Bayesian Information Criterion (BIC), and sample-size adjusted BIC (aBIC), with lower values indicating better fit; (2) classification accuracy: Entropy, ranging from 0 to 1, with values closer to 1 indicating more precise classification; and (3) likelihood ratio tests: Lo-Mendell–Rubin (LMR) and bootstrapped likelihood ratio test (BLRT), with *P* < 0.05 indicating that a model with k classes fits better than one with k-1 classes. In determining the optimal number of profiles, we prioritized a holistic evaluation rather than relying on a single criterion.

Mediation analyses were performed in SPSS 28.0 to examine the mechanism by which FoP affects PD via SOC, using a moderated mediation model. The independent variable was FoP profile, the mediator was SOC score, and the dependent variable was PD. Path coefficients were estimated using partial least squares, and indirect effects were tested with 5000 bootstrap samples to calculate 95% confidence intervals. Analyses were conducted within FoP profiles to explore differences in mediation effects. Statistical significance was set at *P* < 0.05 in two-tailed tests.

## Results

### Demographic characteristics of children with cancer and their parents

A total of 273 parents of children with cancer completed the survey. Over one-third of the children were diagnosed with leukemia (40.66%), and more than half of the parents were aged 30–40 years (53.85%). Additionally, the majority of children were aged 6–14 years (61.90%). Detailed demographic characteristics of the participants are presented in Table 2.

**Table 2:** Demographic characteristics and univariate analysis of latent profiles (*N* = 273).

Items		Overall (*n* = 273) *n* (%)	C1 (*n* = 103) *n* (%)	C2 (*n* = 56) *n* (%)	C3 (*n* = 114) *n* (%)	Value	*P* value
Disease-related items							
Diagnosis (child)	Leukemia	110 (40.29)	48 (46.60)	18 (32.14)	44 (38.60)	16.801^a^	0.065
	Brain and central nervous system tumors	49 (17.95)	13 (12.62)	8 (14.29)	28 (24.56)		
	Lymphoma	37 (13.55)	14 (13.59)	12 (21.43)	11 (9.65)		
	Bone and soft tissue sarcomas	41 (15.02)	12 (11.65)	8 (14.29)	21 (18.42)		
	Solid tumors	27 (9.89)	11 (10.68)	9 (16.07)	7 (6.14)		
	Others	9 (3.29)	5 (4.85)	1 (1.79)	3 (2.63)		
Treatment duration (child)	< 6 months	118 (43.22)	40 (38.83)	26 (46.43)	52 (45.61)	12.963^b^	0.011
	6 months-1 year	82 (30.04)	25 (24.27)	14 (25.00)	43 (37.72)		
	> 1 year	73 (26.74)	38 (36.89)^d^	16 (28.57)^d,e^	19 (16.67)^e^		
Chronic diseases / infections / complications (child)	Yes	115 (42.12)	46 (44.66)	19 (33.93)	50 (43.86)	1.955^b^	0.376
	None	158 (57.88)	57 (55.34)	37 (66.07)	64 (56.14)		
BMI (child)	Low	130 (47.62)	44 (42.72)	29 (51.79)	57 (50.00)	4.423^b^	0.352
	Medium	99 (36.26)	38 (36.89)	17 (30.36)	44 (38.60)		
	High	44 (16.12)	21 (20.39)	10 (17.86)	13 (11.40)		
Anemia (child)	Yes	114 (41.76)	46 (44.66)	26 (46.43)	42 (36.84)	1.992^b^	0.369
	None	159 (58.24)	57 (55.34)	30 (53.57)	72 (63.16)		
History of surgery (child)	Yes	69 (25.27)	11 (10.68)^d^	14 (25.00)^d,e^	44 (38.60)^e^	22.331^b^	< 0.001
	None	204 (74.73)	92 (89.32)^d^	42 (75.00)^d,e^	70 (61.40)^e^		
History of hematopoietic stem cell transplantation (child)	Yes	42 (15.38)	7 (6.80)^d^	10 (17.86)^d,e^	25 (21.93)^e^	9.851^b^	0.007
	None	231 (84.62)	96 (93.20)^d^	46 (82.14)^d,e^	89 (78.07)^e^		
Radiotherapy history^a^ (child)	Yes	75 (27.47)	33 (32.04)	20 (35.71)	22 (19.30)	6.810^b^	0.033
	None	198 (72.53)	70 (67.96)	36 (64.29)	92 (80.70)		
Chemotherapy cycle (child)	0–3	95 (34.80)	36 (34.95)	12 (21.43)^d^	47 (41.23)^e^	19.704^b^	0.003
	4–6	84 (30.77)	38 (36.89)	15 (26.79)	31 (27.19)		
	> 7	44 (16.12)	13 (12.62)	9 (16.07)	22 (19.30)		
	None	50 (18.32)	16 (15.53)^d^	20 (35.71)^e^	14 (12.28)^d^		
Recurrence (child)	Yes	45 (16.48)	10 (9.71)^d^	7 (12.50)	28 (24.56)^e^	9.483^b^	0.009
	None	228 (83.52)	93 (90.29)^d^	49 (87.50)	86 (75.44)^e^		
CVAD catheter (child)	Yes	230 (84.25)	90 (87.38)^d^	37 (66.07)^e^	103 (90.35)^d^	17.903^b^	< 0.001
	None	43 (15.75)	13 (12.62)^d^	19 (33.93)^e^	11 (9.65)^d^		
Individual-related items							
Parent' gender	Male	106 (38.83)	42 (40.78)	24 (42.86)	40 (35.09)	1.219^b^	0.544
	Female	167 (61.17)	61 (59.22)	32 (57.14)	74 (64.91)		
Parent' age (years)	< 30	43 (15.75)	14 (13.59)	11 (19.64)	18 (15.79)	4.431^a^	0.617
	30–40	147 (53.85)	56 (54.37)	26 (46.43)	65 (57.02)		
	40–50	60 (21.98)	21 (20.39)	15 (26.79)	24 (21.05)		
	> 50	23 (8.42)	12 (11.65)	4 (7.14)	7 (6.14)		
Child' gender	Boy	135 (49.45)	54 (52.43)	19 (33.93)^e^	62 (54.39)^d^	6.873^b^	0.032
	Girl	138 (50.55)	49 (47.57)	37 (66.07)^e^	52 (45.61)^d^		
Child' age (years)	0–6	73 (26.74)	31 (30.10)	15 (26.79)	27 (23.68)	1.573^b^	0.814
	6–14	169 (61.90)	60 (58.25)	36 (64.29)	73 (64.04)		
	15–18	31 (11.36)	12 (11.65)	5 (8.93)	14 (12.28)		
Residence	Rural	74 (27.11)	27 (26.21)	20 (35.71)	27 (23.68)	9.113^b^	0.167
	Township	51 (18.68)	23 (22.33)	8 (14.29)	20 (17.54)		
	County town	80 (29.30)	33 (32.04)	10 (17.86)	37 (32.46)		
	City	68 (24.91)	20 (19.42)	18 (32.14)	30 (26.32)		
Educational level	Illiterate	7 (2.56)	4 (3.88)	3 (5.36)	0 (0.00)	18.591^a^	0.088
	Primary school	56 (20.51)	24 (23.30)	10 (17.86)	22 (19.30)		
	Middle school	62 (22.71)	18 (17.48)	13 (23.21)	31 (27.19)		
	High school	49 (17.95)	18 (17.48)	5 (8.93)	26 (22.81)		
	Technical secondary school	47 (17.22)	19 (18.45)	13 (23.21)	15 (13.16)		
	Junior college	29 (10.62)	9 (8.74)	9 (16.07)	11 (9.65)		
	Bachelor's degree or above	23 (8.42)	11 (10.68)	3 (5.36)	9 (7.89)		
Family monthly income (per person) (¥, CNY)	< 1000	58 (21.25)	26 (25.24)	9 (16.07)	23 (20.18)	8.007^a^	0.426
	1001–3000	80 (29.30)	35 (33.98)	14 (25.00)	31 (27.19)		
	3001–5000	78 (28.57)	21 (20.39)	20 (35.71)	37 (32.46)		
	5001–7000	38 (13.92)	13 (12.62)	10 (17.86)	15 (13.16)		
	> 7001	19 (6.96)	8 (7.77)	3 (5.36)	8 (7.02)		
Interpersonal-related items Occupation	Employees of state-owned enterprises or private companies	51 (18.68)	25 (24.27)	8 (14.29)	18 (15.79)	25.812^a^	0.010
	Employees of government or public institutions	52 (19.05)	27 (26.21)^d^	10 (17.86)	15 (13.16)^e^		
	Farmer	53 (19.41)	21 (20.39)	11 (19.64)	21 (18.42)		
	Self-employed	31 (11.36)	9 (8.74)	7 (12.50)	15 (13.16)		
	Retired	15 (5.49)	7 (6.80)	1 (1.79)	7 (6.14)		
	Unemployed	42 (15.38)	7 (6.80)^d^	8 (14.29)	27 (23.68)^e^		
	Other	29 (10.62)	7 (6.80)^d^	11 (19.64)^e^	11 (9.65)		
Social support	Low	100 (36.63)	59 (57.28)^d^	18 (32.14)^e^	23 (20.18)^e^	43.629^b^	< 0.001
	Medium	96 (35.16)	27 (26.21)^d^	28 (50.00)^e^	41 (35.96)		
	High	77 (28.21)	17 (16.50)^d^	10 (17.86)^d^	50 (43.86)^e^		
Self-disclosure	Low	113 (41.39)	22 (21.36)^d^	20 (35.71)^d^	71 (62.28)^e^	51.655^b^	< 0.001
	Medium	71 (26.01)	25 (24.27)	18 (32.14)	28 (24.56)		
	High	89 (32.60)	56 (54.37)^d^	18 (32.14)^e^	15 (13.16)^f^		
Organizational-related items							
Perceived communication quality	Low	70 (25.64)	21 (20.39)	12 (21.43)	37 (32.46)	5.608^b^	0.233
	Medium	71 (26.01)	30 (29.13)	17 (30.36)	24 (21.05)		
	High	132 (48.35)	52 (50.49)	27 (48.21)	53 (46.49)		
Received social work services (financial assistance)	Yes	67 (24.54)	28 (27.18)	10 (17.86)	29 (25.44)	1.789^b^	0.409
	None	206 (75.46)	75 (72.82)	46 (82.14)	85 (74.56)		
Employment status	On the job	92 (33.70)	42 (40.78)	18 (32.14)	32 (28.07)	12.081^b^	0.017
	Resignation	141 (51.65)	36 (34.95)^d^	29 (51.79)	66 (57.89)^e^		
	Take a leave	40 (14.65)	25 (24.27)	9 (16.07)	16 (14.04)		

HMs, hematologic malignancies; CAR-T, Chimeric Antigen Receptor T-cell. C1, Medication Sensitive with Low Fear; C2, Treatment Sensitive with Moderate Fear; C3, Overall High Fear.

a: Fisher's Exact Test. b: Chi-square value. c: CNY=Chinese Yuan, US$1.00 = ¥6.96 (on 2026.1.23).

d, e, f: Values in the same row sharing a common superscript letter (d, e, f) are not significantly different at *P* < 0.05 based on Bonferroni multiple comparisons.

a Although no statistically significant differences were observed for radiotherapy history in multiple comparisons, an overall trend toward group differences was noted. Given the well-documented impact of radiotherapy on disease burden and psychosocial stress among pediatric cancer caregivers,^41^ radiotherapy history was retained as a relevant variable in subsequent analyses.

### Scores of FoP, SOC, and PD

Among parents of children with cancer, the mean score of FoP was 40.27 ± 11.76, with 68% (168/273) exceeding the normal range. The mean SOC score was 58.00 ± 19.27, with 85% (232/273) of parents at medium or low levels. The mean PD score was 27.99 ± 10.85, with 66% (180/273) of parents at medium or high levels (Table 3).

**Table 3:** Scores of FoP, SOC, and PD (*N* = 273).

Variable	Overall	C1: Medication sensitive with low fear	C2: Treatment sensitive with moderate fear	C3: Overall high fear	Kruskal–Wallis test^a^	*P* value
FoP (mean ± SD)	40.27 ± 11.76	27.35 ± 5.40	40.02 ± 2.99	52.06 ± 3.17	234.769	< 0.001
SOC (mean ± SD)	58.00 ± 19.27	69.61 ± 12.79	51.45 ± 21.57	50.79 ± 18.05	59.300	< 0.001
PD (mean ± SD)	27.99 ± 10.85	18.64 ± 4.94	28.39 ± 10.45	36.18 ± 7.83	147.954	< 0.001

SD, standard deviation.

a Equal variances not assumed.

### Latent profile analysis of FoP among parents of children with cancer

Using the 12 items of the FoP-Q-SF/PR as observed indicators, five models were fitted, and the model fit indices are shown in Table 4. As the number of classes increased, the values of AIC, BIC, and aBIC decreased. The entropy value was highest for the three-class model, followed by the two-class model. When the number of classes was set to four or five, the LMR (*P*) values exceeded 0.05, indicating poorer model fit. In contrast, the three-class model showed good fit indices, with both LMR and BLRT (*P*) values < 0.05, suggesting that the three-class solution was the optimal model (Table 4).

**Table 4:** Latent profile analysis model fit metrics.

Class	AIC	BIC	aBIC	Entropy	LMR (*P*)	BLRT (*P*)	Proportion
1	10944.92	11031.549	10955.520	–	–	–	
2	9316.572	9450.122	9332.804	0.944	0.000	0.000	0.46/0.54
3	9001.894	9182.368	9023.830	0.959	0.000	0.000	0.38/0.21/0.41
4	8923.605	9151.001	8951.244	0.930	0.005	0.000	0.24/0.19/0.15/0.41
5	8870.799	9145.118	8904.141	0.945	0.220	0.000	0.12/0.03/0.24/0.41/0.19

AIC, Akaike Information Criterion; BIC, Bayesian Information Criterion; aBIC, adjusted Bayesian Information Criterion; LMRT, Lo-Mendell–Rubin Test; BLRT, Bootstrap Likelihood Ratio Test.

**Fig. 2:**
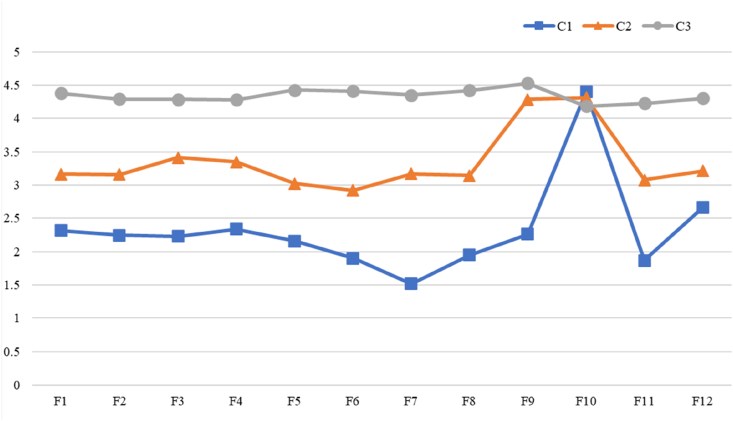
LPA of FoP in parents of children with cancer (C1 = Medication Sensitive with Low Fear; C2 = Treatment Sensitive with Moderate Fear; C3 = Overall High Fear. F1 = I become anxious when I think that my child's disease may progress; F2 = I am nervous prior to doctor's appointments or periodic examinations; F3 = I am afraid that my child may have pain; F4I have concerns about my child being less productive at school because of his/her illness; F5 = When I am anxious, I have physical symptoms (e.g., rapid heartbeat, stomach ache, nervousness); F6 = The possibility that my child may pass the disease on to his/her children disturbs me; F7 = It disturbs me that my child may have to rely on strangers for activities of daily living; F8 = I am worried that at some point in time my child will no longer be able to pursue his/her hobbies because of the illness; F9 = I am afraid of severe medical treatments in the course of my child's illness; F10 = I worry that the medications could damage my child's body; F11 = I worry about what will become of my family if something should happen to my child; F12 = The thought that my child might be absent from school because of his/her illness disturbs me).

Based on the item scores of the three-class model, the distribution characteristics of parental FoP were plotted (Fig. 2). Class 1 demonstrated the lowest overall scores (mostly around 2, with some between 3 and 4), indicating generally low levels of worry, except for a notably high score on “I worry that the medications could damage my child's body” (F10 = 4.4). Class 2 showed moderate overall scores (mostly around 3, with F9 close to 4.3), reflecting moderate levels of worry, particularly regarding “I am afraid of severe medical treatments in the course of my child's illness.” Class 3 exhibited the highest overall scores (almost all between 4.2 and 4.5), indicating high levels of worry across all items. Therefore, the three profiles were labeled as follows: C1: Medication Sensitive with Low Fear (*n* = 103, 38%), C2: Treatment Sensitive with Moderate Fear (*n* = 56, 21%), and C3: Overall High Fear (*n* = 114, 41%).

### Univariate analysis of FoP profiles among parents of children with cancer

Univariate analysis was conducted to compare the general characteristics across the three latent profiles of FoP among parents of children with cancer. The results showed that significant differences existed among the three profiles in terms of treatment duration (child), history of surgery (child), history of hematopoietic stem cell transplantation (child), radiotherapy history (child), chemotherapy cycle (child), recurrence (child), CVAD catheter (child), child's gender, occupation, social support, self-disclosure, and employment status. The results are presented in Table 2.

### Multivariate analysis of FoP profiles among parents of children with cancer

Variables with statistically significant differences in the univariate analysis were included as independent variables (after testing for multicollinearity, all VIF values were < 5). The three FoP profiles identified by LPA were set as dependent variables, and Logistic regression was performed. In the model construction, the C1 profile was first used as the reference category and compared separately with the C2 and C3 profiles; subsequently, the C2 profile was used as the reference category and compared with the C3 profile. The results of the Logistic regression analysis are presented in Table 5.

**Table 5:** Multivariate Logistic regression of Latent profiles.

Reference group		Medication Sensitive with Low Fear						Treatment Sensitive with Moderate Fear		
Comparison group		Treatment Sensitive with Moderate Fear			Overall High Fear			Overall High Fear		
		OR	95% CI	*P value*	OR	95% CI	*P value*	OR	95% CI	*P value*
Treatment duration (child)	< 6 months	0.879	0.251–3.085	0.841	3.860	0.982–15.173	0.053	4.390	1.083–17.799	0.038
	6 months-1 year	1.935	0.607–6.167	0.265	7.018	1.982–24.848	0.003	3.628	1.030–12.781	0.045
	> 1 year	0^b^			0^b^			0^b^		
History of surgery (child)	Yes	6.178	1.903–20.057	0.002	12.336	3.889–39.124	0.000	1.997	0.789–5.054	0.144
	None	0^b^			0^b^			0^b^		
History of hematopoietic stem cell transplantation (child)	Yes	3.636	1.034–12.785	0.044	5.404	1.536–19.008	0.009	1.486	0.482–4.580	0.490
	None	0^b^			0^b^			0^b^		
Radiotherapy history (child)	Yes	1.154	0.453–2.940	0.764	1.012	0.369–2.779	0.981	0.877	0.325–2.368	0.796
	None	0^b^			0^b^			0^b^		
Chemotherapy cycle (child)	0–3	0.872	0.052–14.675	0.925	0.777	0.054–11.098	0.852	0.890	0.063–12.606	0.932
	4–6	1.063	0.064–17.767	0.966	0.758	0.054–10.662	0.837	0.713	0.049–10.400	0.805
	> 7	0.957	0.052–17.678	0.976	1.012	0.066–15.525	0.993	1.058	0.068–16.543	0.968
	None	0^b^			0^b^			0^b^		
Recurrence (child)	Yes	0.927	0.238–3.616	0.913	3.354	0.863–13.041	0.081	3.617	1.028–12.723	0.045
	None	0^b^			0^b^			0^b^		
CVAD catheter (child)	Yes	0.263	0.014–4.968	0.373	2.875	0.162–51.000	0.472	10.952	0.654–183.307	0.096
	None	0^b^			0^b^			0^b^		
Child' gender	Boy	0.418	0.178–0.984	0.046	1.045	0.453–2.413	0.917	2.499	1.074–5.813	0.033
	Girl	0^b^			0^b^			0^b^		
Occupation	Employees of state-owned enterprises or private companies	0.150	0.030–0.742	0.020	0.161	0.027–0.979	0.047	1.072	0.229–5.013	0.930
	Employees of government or public institutions	0.096	0.019–0.474	0.004	0.096	0.016–0.573	0.010	1.003	0.226–4.448	0.997
	Farmers	0.230	0.048–1.111	0.067	0.219	0.037–1.279	0.092	0.951	0.223–4.064	0.946
	Self-employed individuals	0.296	0.051–1.712	0.174	0.635	0.092–4.376	0.645	2.148	0.424–10.881	0.356
	Retired	0.029	0.002–0.489	0.014	0.207	0.017–2.498	0.215	7.188	0.481–107.351	0.153
	Unemployed	0.320	0.053–1.928	0.214	0.700	0.108–4.527	0.708	2.187	0.494–9.673	0.302
	Other	0^b^			0^b^			0^b^		
Social support	Low	0.415	0.127–1.358	0.146	0.064	0.020–0.209	0.000	0.154	0.045–0.526	0.003
	Medium	2.107	0.620–7.169	0.233	0.348	0.111–1.087	0.069	0.165	0.053–0.516	0.002
	High	0^b^			0^b^			0^b^		
Self-disclosure	Low	4.661	1.632–13.313	0.004	56.026	16.232–193.385	0.000	12.020	3.638–39.716	0.000
	Medium	1.967	0.679–5.694	0.212	13.818	3.994–47.806	0.000	7.026	1.994–24.756	0.002
	High	0^b^			0^b^			0^b^		
Employment status	On the job	1.092	0.352–3.385	0.879	0.463	0.145–1.472	0.192	0.424	0.122–1.466	0.175
	Resignation	2.586	0.818–8.183	0.106	1.817	0.625–5.284	0.273	0.703	0.222–2.218	0.547
	Take a leave	0^b^			0^b^		0	0^b^		

CVAD: Central Venous Access Device.

### Mediation testing

#### Correlation analysis

The results indicated that FoP among parents of children with cancer was negatively correlated with SOC (*r* = −0.525, *P* < 0.001) and positively correlated with PD (*r* = 0.749, *P* < 0.001). In addition, SOC was negatively correlated with PD (*r* = −0.669, *P* < 0.001), as shown in Table 6.

**Table 6:** Correlation analysis of FoP, SOC and PD.

Items	FoP	SOC	PD
FoP	1.000		
SOC	−0.525^a^	1.000	
PD	0.749^a^	−0.669^a^	1.000

FoP, Fear of Progression; SOC, Sense of Coherence; PD, Psychological Distress.

a *P* < 0.001.

#### Mediation analysis

To explore the underlying mechanism of the significant positive effect of FoP on PD, SOC was introduced as a mediator into the structural equation model. Mediation testing was conducted using Model 4 of the PROCESS macro in SPSS. Path analyses were performed for the overall sample as well as for the three latent profiles. The mediation effect of SOC between FoP and PD was further examined using Hayes' bootstrapping method. Specifically, FoP was negatively associated with SOC (path a), whereas SOC was negatively associated with PD after controlling for FoP (path b), indicating a potential buffering role of SOC. The direct effect of FoP on PD (path c’) remained significant in the overall sample and in “treatment sensitive with moderate fear” and “overall high fear”.

**Fig. 3:**
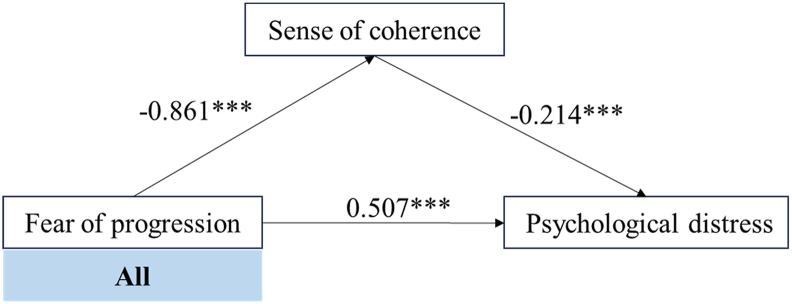
Mediation Effect Analysis of the overall participants. ∗∗∗*P* < 0.001.

**Fig. 4:**
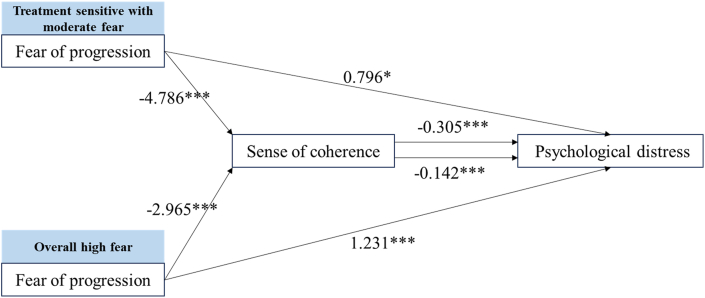
Mediation effect analysis of the FoP profiles. ∗∗∗*P* < 0.001, ∗∗*P* < 0.01, ∗*P* < 0.05. FoP, Fear of Progression.

The standardized path coefficients for each mediation pathway (FoP → SOC, SOC → PD, and FoP → PD) are presented in (Fig. 3: overall sample; Fig. 4: “treatment sensitive with moderate fear” and “overall high fear”). The mediation effect was not supported in the “medication sensitive with low fear”.

As shown in Table 7, the bootstrap 95% confidence intervals for both the direct and indirect effects of FoP on PD through SOC in the overall sample, “treatment sensitive with moderate fear”, and “overall high fear” did not contain zero, indicating that FoP exerted not only a direct effect on PD but also an indirect effect via SOC. The magnitude of the indirect effect was particularly pronounced in the “treatment sensitive with moderate fear”, suggesting a stronger buffering effect of SOC in this profile.

**Table 7:** Total effect, direct effect and indirect effect decomposition table.

Variables		Effect	SE	LLCI	ULCI	
All	Total effect	0.692	0.371	0.619	0.765	/
	Direct effect	0.507	0.038	0.432	0.582	73.27%
	Indirect effect	0.184	0.027	0.135	0.239	26.59%
Medication sensitive with low fear	Total effect	−0.133	0.090	−0.312	0.046	/
	Direct effect	−0.177	0.091	−0.358	0.004	132.86%
	Indirect effect	0.044	0.024	0.001	0.097	−32.93%
Treatment sensitive with moderate fear	Total effect	2.257	0.362	1.531	2.983	/
	Direct effect	0.796	0.385	0.024	1.568	35.27%
	Indirect effect	1.461	0.280	0.917	2.025	64.73%
Overall high fear	Total effect	1.653	0.174	1.308	1.997	/
	Direct effect	1.232	0.190	0.856	1.607	74.53%
	Indirect effect	0.421	0.137	0.178	0.720	25.47%

SE, Standard error; LLCI, lower level of confidence interval; ULCI, upper level of confidence interval.

## Discussion

### Main findings

This study explored the FoP in parents of children with cancer, a topic that has been insufficiently examined in the existing literature. To the best of our knowledge, this is the first study to use LPA to systematically investigate FoP heterogeneity among parents of children with cancer within a Chinese population.

The mean FoP score in our sample (40.27 ± 11.74) was higher than that reported in studies of parents of children with cancer in Germany (33.8 ± 9.4)^16^ and those with retinoblastoma in China (37.06 ± 9.03).^6^ This elevated FoP may be attributable to two primary factors: the heterogeneity of the cancer diagnoses in our study, which included a wide range of prognoses and treatment complexities,^4^^,^^12^ and sociocultural factors in China, such as the internalization of parental responsibility^42^ and the suppression of negative emotions.^43^ This psychological burden is further compounded by our finding that a majority of parents (67.6%) reported low to moderate levels of self-disclosure. This reluctance to share distress, possibly also influenced by cultural norms, may create a cycle where unexpressed fear intensifies internal PD.^44^ The mean SOC score was 58.00 ± 19.27, which can be considered at a moderate-to-low level, yet still higher than that reported among parents in southeastern Iran (51.4 ± 14.2).^29^ This difference may be attributable to contextual and resource-related factors. The current sample was recruited from provincial-level hospitals with relatively higher economic and medical resources, where parents had greater access to standardized diagnosis, treatment, and information support, which may have contributed to enhancing their SOC.^30^ The mean PD score was 27.99 ± 10.85. Previous studies have shown that parents of children with cancer generally report elevated levels of PD, anxiety, and depression,^45^, ^46^, ^47^ indicates that the long-term stress and uncertainty during the care process have had a significant impact on the parents' mental health.^6^^,^^12^

This study identified three latent profiles of FoP among parents through LPA: the “medication sensitive with low fear” (38%), the “treatment sensitive with moderate fear” (21%), and the “overall high fear” (41%). This finding clearly demonstrates that parents exhibit substantial heterogeneity in their responses to the progression of their child's disease. Notably, the proportion of the “overall high fear” profile in our sample (41%) is markedly higher than the “severe fear” profile (17%) reported in a study of adult colorectal cancer patients.^48^ We propose that this heightened prevalence and complexity of fear profiles can be attributed to a combination of disease-specific and sociocultural factors. First, the nature of pediatric malignancies inherently generates greater uncertainty. Compared to many adult cancers, childhood cancers are diverse,^1^ often requiring prolonged, complex treatment regimens with considerable uncertainty regarding recurrence and long-term side effects.^49^ This context of intensive caregiving responsibilities coupled with profound outcome uncertainty likely elevates the baseline level and complexity of parental fear.^6^^,^^50^ Second, specific sociocultural factors in China may further exacerbate these anxieties. Sociological studies have consistently highlighted the profound stigma and fatalism associated with a cancer diagnosis in Chinese society, which can amplify parents' existential fears.^51^ This is often compounded by strong, culturally ingrained parental expectations for their children's future, where a child's illness is perceived as a catastrophic threat to family continuity.^11^^,^^52^ Interestingly, our own findings provide nuanced support for this cultural interpretation. In the current study, participants in the “overall high fear” profile exhibited higher perceived social support but significantly lower self-disclosure scores compared to the “treatment sensitive with moderate fear” profile.^52^ Although individuals may receive external support, they may simultaneously suppress their own emotional distress to preserve social harmony. Such culturally influenced emotional suppression may, in turn, intensify and internalize FoP.

In addition, when compared with findings from Chinese survivors of hematologic malignancies, in which three profiles were identified: the low-risk fear group (20.88%), the moderate-risk fear group (54.73%), and the high-risk fear group (24.49%),^25^ the present study showed a higher proportion of parents in the comprehensive high–fear profile (41%) and a relatively lower proportion in the moderate–fear profile. This difference may be attributed to variations in disease stage and role characteristics. Survivors, having completed the major phases of treatment, although still at risk of recurrence, may experience moderate levels of fear.^3^^,^^53^ By contrast, as Peikert's research shows, parents of pediatric patients are engaged throughout the entire trajectory of diagnosis, treatment, and long-term caregiving.^16^ They not only endure the demands of caregiving and financial burden but also face uncertainty about their child's future development, which may contribute to their higher levels of FoP.^16^

Building on the LPA of parental FoP, this study further conducted multifactor pairwise comparisons to examine factors associated with profiles. The results indicated that differences in parental FoP were closely related to children's disease characteristics as well as family and social environments. Specifically, compared with parents in “medication sensitive with low fear” profile, those whose children had a history of surgery or hematopoietic stem cell transplantation, whose child was female, whose occupation was neither in state-owned or private enterprises nor in government or public institutions, or who were retired, and those who reported lower levels of self-disclosure were more likely to be classified into “treatment sensitive with moderate fear” profile. This finding suggests that parents of children who undergo more complex treatments or major medical events are more prone to moderate levels of FoP, possibly due to heightened uncertainty and information burden associated with treatment experiences.^16^ Consistent with this, Christen et al.^53^ reported that more than half of parents experienced elevated levels of worry and anxiety due to ongoing disadvantages caused by their child's previous illness, while Collins et al.^54^ found that parents of children with multifocal retinoblastoma had a higher prevalence of depression than those of children with unifocal disease. Moreover, parents in non-formal or unstable occupations may have fewer social resources and less adaptive capacity, thereby increasing their vulnerability to disease-related concerns. Parents with lower self-disclosure may be less inclined to express emotions or seek help,^55^ which weakens the buffering effect of social support and contributes to a higher likelihood of experiencing moderate levels of worry when facing disease progression.^56^

Compared with “medication sensitive with low fear” profile, parents in the “overall high fear” profile reported higher levels of FoP. Parents whose children had been in treatment for less than six months, had undergone surgery or hematopoietic stem cell transplantation, whose occupations were outside state-owned or private enterprises or government/public institutions, and those who perceived high social support but reported low self-disclosure were more likely to be classified into “overall high fear type” profile. This suggests that the early treatment stage and major medical interventions may trigger more pervasive parental concerns. Although these parents perceived a certain level of social support, their limited self-disclosure may have restricted their ability to effectively utilize these resources to alleviate anxiety.^56^ Even when family or social support is available, the absence of emotional expression or help-seeking behaviors may prevent a substantial reduction in psychological burden.^56^ Fang et al.^37^ also demonstrated that self-disclosure could influence caregivers’ PD both directly and indirectly through perceived social support. In the current study, participants in the “overall high fear” profile exhibited higher perceived social support but lower self-disclosure scores compared with the “medication sensitive with low fear” profile, indicating that although these parents received support from their social networks, they tended to suppress or under-report personal distress, which may contribute to elevated FoP.

When comparing “overall high fear” profile with “treatment sensitive with moderate fear” profile, parents whose children had been in treatment for less than one year, had experienced relapse, were male, and whose parents perceived high social support but low self-disclosure were more likely to be assigned to “overall high fear” profile. This finding highlights that in the early phase of treatment or in the case of relapse, even when parents perceive adequate social support, a lack of self-disclosure may hinder their ability to seek and use assistance, thereby contributing to heightened levels of worry.^44^ Moreover, the higher FoP observed among parents of male children may reflect cultural expectations in China, where parents are often more sensitive to the suffering of boys or the potential impact of illness on their future development.^11^ In contrast, in the comparison between “treatment sensitive with moderate fear” profile and “medication sensitive with low fear” profile, parents of female children reported higher FoP, which may be related to the limited sample size and the single-site design of this study in a provincial hospital. This suggests that further research with larger, multicenter samples is needed to verify potential gender differences.

### Implications for nursing practice and research

The findings of this study carry significant clinical implications for psychosocial screening and intervention. The high proportion of parents classified into the moderate- and high–FoP profiles underscores that routine screening for FoP is clinically warranted. Such screening should aim not only to identify the level of fear but also its specific pattern, enabling a shift from one-size-fits-all support to precision intervention. A central finding of this research is the critical, protective role of SOC. Our mediation analyses demonstrated that for parents in the “treatment sensitive with moderate fear” and “overall high fear” profiles, the detrimental effect of FoP on PD was significantly mediated by SOC.^16^^,^^28^ This indicates that SOC acts as a powerful buffer, particularly for parents experiencing moderate to high levels of concern. This result aligns with the Transactional Stress Model,^27^ which posits that stable psychological resources like SOC can improve cognitive appraisal and coping, thereby mitigating distress in the face of ongoing stressors.^27^^,^^28^^,^^30^ Taken together, these findings highlight that SOC should be a key target in interventions aimed at improving parental mental health.

From an intervention perspective, our results suggest that parents in the “overall high fear” profile would particularly benefit from SOC-oriented strategies.^28^^,^^29^ These could include psychoeducation focused on enhancing illness comprehensibility, cognitive-behavioral techniques to bolster perceived manageability, and meaning-centered therapies to strengthen a sense of purpose.^29^^,^^30^^,^^57^ Therefore, incorporating FoP-based profile screening into routine clinical practice would allow for the efficient allocation of psychosocial resources, ensuring that parents at the greatest psychological risk receive targeted, SOC-enhancing support, while those with low concern receive support focused on maintaining their existing resilience. Future research could further refine this precision approach by exploring the use of machine learning models to predict FoP profiles, thereby advancing risk stratification and the development of truly personalized interventions.^8^ We plan to use the identified FoP profiles as the outcome variable. By combining these profiles with key indicators readily available in clinical practice, we aim to develop a rapid predictive model for FoP profiles using machine learning methods.^8^ This model is expected to support clinicians in conducting preliminary FoP risk stratification for parents within limited time constraints, thereby enabling the matching of appropriate intervention strategies.

### Limitations

Several limitations should be acknowledged in this study. First, the cross-sectional design restricts the ability to draw causal inferences between FoP, SOC, and PD. Future longitudinal studies are warranted to clarify the dynamic and temporal relationships among these variables. Second, the study sample was drawn using a convenience sampling method from three provincial-level hospitals in Changsha, Hunan province, which introduces several potential limitations. This non-probability sampling strategy may have led to selection bias, as the recruited sample might not be fully representative of the broader population of parents of children with cancer. Furthermore, recruiting from only three tertiary hospitals may limit the generalizability of the findings, the parents were caring for children with different types of cancer, which may vary substantially in terms of disease trajectory, treatment intensity, prognosis, and caregiving demands. Such heterogeneity across cancer types may influence parents’ psychological experiences and the developmental trajectories of FoP, SOC, and PD, and was not explicitly accounted for in the present analysis. Meanwhile, the experiences and resources of parents from rural areas, community-level hospitals, or other provinces with different socioeconomic and cultural backgrounds may not be adequately captured. Future research should employ probability-based sampling strategies across multiple geographic regions (including rural and urban settings, different cultural groups) and consider stratification or subgroup analyses by cancer type to enhance the external validity and confirm the stability of our findings. Finally, the LPA was conducted with a moderate sample size; replication in larger, multi-center cohorts is necessary to confirm the stability and robustness of the identified subtypes.

## Conclusions

This study identified three distinct latent profiles of FoP among parents of children with cancer: “medication sensitive with low fear”, “treatment sensitive with moderate fear”, and “overall high fear”, which highlight significant individual differences in parental psychological responses. SOC was found to partially mediate the relationship between FoP and PD in parents with moderate and high fear, suggesting that stable psychological resources can buffer the negative impact of disease-related worries. From a clinical perspective, these findings support the use of FoP profile identification as a practical screening and risk stratification tool in pediatric oncology settings. Parents characterized by moderate or high FoP profiles may benefit from early psychological assessment and prioritized psychosocial support. Moreover, SOC represents a modifiable and clinically meaningful intervention target. Interventions aimed at enhancing comprehensibility, manageability, and meaningfulness, such as structured psychoeducation, cognitive-behavioral strategies, and family-centered support, may be particularly effective in reducing anxiety and depression among high-risk parents. Incorporating FoP profile assessment and SOC-enhancing strategies into routine clinical practice may facilitate more individualized, resource-efficient psychosocial care, ultimately improving psychological outcomes for both parents and their children.

## CRediT authorship contribution statement

**Guiyuan Ma:** Conceptualization, Methodology, Writing – Original Draft. **Peijuan Jiao**: Data Curation, Formal Analysis, Visualization. **Xiaorou Zeng**: Data Curation, Formal Analysis, Resources, Supervision. **Qiming Ding**: Data Curation, Supervision. **Zhixiang Huang**: Software, Validation. **Can Gu and Jianhui Xie:** Writing – Review & Editing, Project Administration, Funding Acquisition. All authors have read and approved the final manuscript.

## Ethics statement

The study was approved by the Medical Ethics Committee of Hunan Children's Hospital (Approval No. HCHLL-2025-289) and was conducted in accordance with the 1964 Helsinki Declaration and its later amendments or comparable ethical standards. All participants provided written informed consent.

## Data availability statement

The datasets analyzed during the current study are available from the corresponding author upon reasonable request. In line with open science and data sharing principles, the authors support responsible data sharing. Due to ethical restrictions and the sensitive nature of the data involving parents of pediatric cancer patients, the datasets are not publicly available. However, de-identified data can be shared with qualified researchers upon reasonable request, subject to approval by the institutional ethics committee.

## Declaration of generative AI and AI-assisted technologies in the writing process

No AI tools/services were used during the preparation of this work.

## Funding

This study was supported by the National Nature Science Foundation in China (Grant No. 82272924), Chia Community Health Services Program (Grant No. 2023YC01), and Hunan Provincial Innovation Foundation for Postgraduate (Grant No. CX20240325). The funders had no role in considering the study design or in the collection, analysis, interpretation of data, writing of the report, or decision to submit the article for publication.

## Declaration of competing interest

The authors declare no conflict of interest. The corresponding author, Prof. Gu Can, is an editorial board member of *Asia–Pacific Journal of Oncology Nursing*. The article was subject to the journal's standard procedures, with peer review handled independently of Prof. Gu Can and their research groups.
