# Supplementary material for: Fear of progression profiles in parents of children with cancer and their impact on psychological distress: The mediating role of sense of coherence
Source: Asia Pac J Oncol Nurs. 2026 Mar 3;13:100926. doi: 10.1016/j.apjon.2026.100926 (PMC13054603; doi:10.1016/j.apjon.2026.100926)
Supplement: Multimedia component 1 [file mmc1.docx]

Respondent ID: Survey Date: Year Month Day

Dear Parent,

Thank you for taking the time to participate in this study. This questionnaire aims to understand the feelings, psychological experiences, and support received by parents of children with cancer during the caregiving process. The findings will provide scientific basis for developing more targeted psychological support and services in the future.

This questionnaire is strictly for research purposes. All information will be kept confidential and analyzed anonymously. It will not affect your treatment or follow-up care in any way. There are no “right” or “wrong” answers. Please respond based on your genuine feelings.

Completing this questionnaire will take approximately 15–25 minutes. You may withdraw at any time without any consequences.

Thank you again for your understanding and support. Wishing you and your child all the best!

**Demographic and Disease Information**

**Part I (This section collects your basic information. Please fill in the corresponding spaces or mark “√” at the appropriate number)**

1. Your age: Your gender:
2. Your child's age: Your child's gender:
3. Place of residence: □ Rural □ Township □ County town □ City
4. Family monthly income (per person): □ Below ¥1,000 □ ¥1,001–3,000 □ ¥3,001–5,000 □ ¥5,001–7,000 □ ¥7,001 and above
5. Your educational level: □ Illiterate □ Primary school □ Middle School □ High School □ Technical Secondary School □ Junior College □ Bachelor's degree or higher
6. Your occupation: □ Employee of state-owned or private companies □ Employee of government or public institution (e.g., teacher, civil servant, healthcare worker) □ Farmer □ Self-employed □ Retired □ Unemployed □ Other (please specify)
7. Your employment status: □ On the job □ Resignation □ Take a leave
8. Primary diagnosis of your child's illness:
9. Your child's treatment duration: □ <6 months □ 6 months~ 1 year □ 1 year or longer
10. Presence of other chronic diseases/infections/complications (your child): □ None □ Yes
11. Your child's height: cm Weight: kg
12. Does your child have anemia: □ No □ Yes
13. Does your child have a history of surgery: □ No □ Yes
14. Does your child have a history of hematopoietic stem cell transplantation: □ No □ Yes
15. Does your child have a history of radiation therapy: □ No □ Yes
16. Your child is currently in the chemotherapy phase:
17. Has your child's illness ever recurred? □ No □ Yes
18. Does your child have a central venous access device? □ No □ Yes
19. Perceived communication quality from doctors/nurses: □ High □ Medium □ Low
20. Have you received social work services (financial assistance)? □ Yes □ No

**Part II (Please fill in the corresponding spaces or check the box)**

1. **Fear of Progression Questionnaire-Short Form for Parents (FoP-Q-SF/PR)**

The following questions primarily explore your worries and feelings regarding potential recurrence or progression of the disease during your child's illness and treatment.

Please recall your genuine thoughts from the recent period and select the option that best reflects your current feelings.

These queries do not imply that your child will experience adverse outcomes; they are solely intended to understand parents' psychological experiences.

Please answer freely—there are no standard answers.

|  | My actual experience (feelings) over the past month: | Never | Rarely | Sometimes | Often | Always |
| --- | --- | --- | --- | --- | --- | --- |
| 1 | I become anxious when I think that my child's disease may progress. | 1 | 2 | 3 | 4 | 5 |
| 2 | I am nervous prior to doctor's appointments or periodic examinations. | 1 | 2 | 3 | 4 | 5 |
| 3 | I am afraid that my child may have pain. | 1 | 2 | 3 | 4 | 5 |
| 4 | I have concerns about my child being less productive at school because of his/her illness. | 1 | 2 | 3 | 4 | 5 |
| 5 | When I am anxious, I have physical symptoms (e.g. rapid heartbeat, stomach ache, nervousness). | 1 | 2 | 3 | 4 | 5 |
| 6 | The possibility that my child may pass the disease on to his/her children disturbs me. | 1 | 2 | 3 | 4 | 5 |
| 7 | It disturbs me that my child may have to rely on strangers for activities of daily living. | 1 | 2 | 3 | 4 | 5 |
| 8 | I am worried that at some point in time my child will no longer be able to pursue his/her hobbies because of the illness. | 1 | 2 | 3 | 4 | 5 |
| 9 | I am afraid of severe medical treatments in the course of my child's illness. | 1 | 2 | 3 | 4 | 5 |
| 10 | I worry that the medications could damage my child's body. | 1 | 2 | 3 | 4 | 5 |
| 11 | I worry about what will become of my family if something should happen to my child. | 1 | 2 | 3 | 4 | 5 |
| 12 | The thought that my child might be absent from school because of his/her illness disturbs me. | 1 | 2 | 3 | 4 | 5 |

1. **Kessler Psychological Distress Scale (K10)**

The following questions are designed to assess your recent psychological state, such as feelings of tension, anxiety, or low mood.

|  | In the past 4 weeks, how often | All the time | Most of the time | Some of the time | A little of the time | None of the time |
| --- | --- | --- | --- | --- | --- | --- |
| 1 | Did you feel tired out for no good reason? | 5 | 4 | 3 | 2 | 1 |
| 2 | Did you feel nervous? | 5 | 4 | 3 | 2 | 1 |
| 3 | Did you feel so nervous that nothing could calm you down? | 5 | 4 | 3 | 2 | 1 |
| 4 | Did you feel hopeless? | 5 | 4 | 3 | 2 | 1 |
| 5 | Did you feel restless or fidgety? | 5 | 4 | 3 | 2 | 1 |
| 6 | Did you feel so restless that you could not sit still? | 5 | 4 | 3 | 2 | 1 |
| 7 | Did you feel depressed? | 5 | 4 | 3 | 2 | 1 |
| 8 | Did you feel that everything was an effort? | 5 | 4 | 3 | 2 | 1 |
| 9 | Did you feel so sad that nothing could cheer you up? | 5 | 4 | 3 | 2 | 1 |
| 10 | Did you feel worthless? | 5 | 4 | 3 | 2 | 1 |

1. **Perceived Social Support Scale (PSSS)**

The following questions primarily explore the support you feel from family, friends, or others while caring for your child. Here, “support” includes emotional care and understanding, as well as assistance with daily life or caregiving tasks.

Please answer based on your current, genuine feelings.

|  | Items | Very Strongly Disagree | Strongly Disagree | Mildly Disagree | Neutral | Mildly Agree | Strongly Agree | Very Strongly Agree |
| --- | --- | --- | --- | --- | --- | --- | --- | --- |
| 1 | When I encounter problems, some people (family members) will be there for me. | 1 | 2 | 3 | 4 | 5 | 6 | 7 |
| 2 | I can share both joy and sorrow with some people (family members). | 1 | 2 | 3 | 4 | 5 | 6 | 7 |
| 3 | My family provides me with tangible, concrete help. | 1 | 2 | 3 | 4 | 5 | 6 | 7 |
| 4 | When needed, I can receive emotional support and assistance from my family. | 1 | 2 | 3 | 4 | 5 | 6 | 7 |
| 5 | I have close friends who offer me support and help. | 1 | 2 | 3 | 4 | 5 | 6 | 7 |
| 6 | I can discuss my difficulties with my friends. | 1 | 2 | 3 | 4 | 5 | 6 | 7 |
| 7 | My friends can share both my joys and sorrows with me. | 1 | 2 | 3 | 4 | 5 | 6 | 7 |
| 8 | When I need it, I can receive emotional help and support from my friends. | 1 | 2 | 3 | 4 | 5 | 6 | 7 |
| 9 | When I encounter problems, certain people (leaders, relatives, colleagues) will be there for me. | 1 | 2 | 3 | 4 | 5 | 6 | 7 |
| 10 | I can share both my joys and sorrows with certain people (leaders, relatives, colleagues). | 1 | 2 | 3 | 4 | 5 | 6 | 7 |
| 11 | In my life, certain people (leaders, relatives, colleagues) care about my emotional well-being. | 1 | 2 | 3 | 4 | 5 | 6 | 7 |
| 12 | When needed, I can receive emotional support and help from certain people (leaders, relatives, colleagues). | 1 | 2 | 3 | 4 | 5 | 6 | 7 |

1. **Sense of Coherence-13 (SOC-13)**

The following questions primarily explore your understanding of situations, coping abilities, and sense of meaning in life when facing stress related to daily living and illness. Please answer based on your usual feelings; there are no right or wrong answers.

|  |  | 1 | 2 | 3 | 4 | 5 | 6 | 7 |
| --- | --- | --- | --- | --- | --- | --- | --- | --- |
| 1 | Do you often feel indifferent to what's happening around you? | Never | Rarely | Seldom | Hard to say | Sometimes | Often | Frequently |
| 2 | Have you frequently experienced situations where people you relied on let you down? | Never | Rarely | Seldom | Hard to say | Sometimes | Often | Frequently |
| 3 | Or where those you thought you knew well surprised you with their actions? | Never | Rarely | Seldom | Hard to say | Sometimes | Often | Frequently |
| 4 | Many people, even those with great talent, sometimes feel like failures under certain circumstances. In your past experiences, have you often felt this way? | Never | Rarely | Seldom | Hard to say | Sometimes | Often | Frequently |
| 5 | Do you frequently feel treated unfairly? | Never | Rarely | Seldom | Hard to say | Sometimes | Often | Frequently |
| 6 | Do you often have very complex, mixed feelings and thoughts? | Very frequently | Often | Sometimes | Not sure | Rarely | Very rarely | Almost never or never |
| 7 | Do you often experience emotions you wish you didn't have? | Very frequently | Often | Sometimes | Not sure | Rarely | Very rarely | Almost never or never |
| 8 | Do you frequently feel out of control? | Very frequently | Often | Sometimes | Not sure | Rarely | Very rarely | Almost never or never |
| 9 | How often do you think the things you do every day lack meaning? | Very frequently | Often | Sometimes | Not sure | Rarely | Very rarely | Almost never or never |
| 10 | So far, your life: | No goals whatsoever | Few goals | Not very purposeful | Can't say for sure | Have life goals | Have relatively clear life goals | Have very clear life goals |
| 11 | Doing the things you do every day is: | Great joy and satisfaction | Fairly happy and satisfied | Somewhat happy | Hard to say | Somewhat unhappy | Very unhappy | A source of pain and distress |
| 12 | When faced with problems or situations, you usually find yourself: | Underestimated or overestimated its importance | Difficult to estimate accurately | Estimated somewhat inaccurately | Hard to grasp | Evaluated this matter relatively accurately | Evaluated this matter accurately | Evaluated it very correctly |
| 13 | Do you often feel like you're in unfamiliar environments where you don't know what to do? | Frequently | Sometimes | Occasionally | Not sure | Rarely | Very rarely | Almost never or never |

1. **Distress Disclosure--** **The Distress Disclosure Assessment Tool (DDAT)**

The following questions are designed to understand how often you share or express these feelings with others when you feel stressed, worried, or emotionally distressed.

Please answer based on your daily habits and genuine thoughts, rather than what you think you “should” do.

|  | Items | Strongly disagree | Somewhat disagree | Undecided | Somewhat agree | Strongly agree |
| --- | --- | --- | --- | --- | --- | --- |
| 1 | When I'm feeling down, I usually confide in friends. | 1 | 2 | 3 | 4 | 5 |
| 2 | I don't like to talk about my problems. | 1 | 2 | 3 | 4 | 5 |
| 3 | When unpleasant things happen to me, I often seek someone to discuss them with. | 1 | 2 | 3 | 4 | 5 |
| 4 | I generally don't discuss things that make me sad with others. | 1 | 2 | 3 | 4 | 5 |
| 5 | When I feel depressed or upset, I always bear it alone. | 1 | 2 | 3 | 4 | 5 |
| 6 | I will talk to someone about my problems. | 1 | 2 | 3 | 4 | 5 |
| 7 | When I'm in a bad mood, I seek out friends to chat with. | 1 | 2 | 3 | 4 | 5 |
| 8 | If I'm feeling down, I'm least inclined to confide in others. | 1 | 2 | 3 | 4 | 5 |
| 9 | When I encounter difficulties, I rarely discuss them with others. | 1 | 2 | 3 | 4 | 5 |
| 10 | When I'm in pain, I won't tell anyone. | 1 | 2 | 3 | 4 | 5 |
| 11 | When I'm feeling low, I usually find someone to talk to. | 1 | 2 | 3 | 4 | 5 |
| 12 | I'm willing to share my unhappiness with others. | 1 | 2 | 3 | 4 | 5 |

If you feel unwell while completing this questionnaire, you may pause or exit at any time and seek assistance from medical staff or mental health support personnel.

Researcher Contact: xxx

Tel: 130xxxxxxxx
